# The Best Hysterias: Author's Response to Ghaemi

**DOI:** 10.1371/journal.pmed.0030320

**Published:** 2006-07-25

**Authors:** David Healy

**Affiliations:** **1**Cardiff UniversityBangorUnited Kingdom

Nassir Ghaemi has helped raise the profile of this truly debilitating disorder [ 1], but he is wrong on the history of bipolar disorder. First, mental disease entities are a recent construct. No disease resembling bipolar disorder was described before 1854 in Paris, and the links between folie circulaire described then and modern bipolar disorder are tenuous. Second, for the Greeks, mania referred to any overactive insanity, and melancholia to any underactive state. The majority of manias were probably delirious states. The melancholias may have been anything from Parkinson disease to hypothyroidism. Third, Emil Kraepelin's manic-depressive insanity (1899) was a very different disorder to bipolar disorder, which only arose in the late 1960s. If bipolar disorder can be clearly traced back to the Greeks, the fact that American physicians so rarely made the diagnosis before 1970—when lithium was introduced in the United States—is hard to explain. Kraepelin's likely response to recent proposals that we recognize and distinguish between bipolar 1, 2, 2.5, 3, 3.5, 4, 5, and 6 and bipolar spectrum disorders would probably not be printable.

Disease mongering is not the creation of diseases de novo, as in the restless legs syndrome Dr Ghaemi cites, descriptions of which go back to antiquity. As so aptly defined by David Menkes at the Conference on Disease Mongering in Newcastle in 2006, disease mongering is where the interests of the seller of a nostrum, who sells by emphasizing the existence of and risks of some condition, in fact outweigh the likely benefits from the proposed remedy to those affected by the putative condition. It shades into hucksterism and it was associated with Harley Street long before modern pharmaceutical companies. But companies now bring an industrial efficiency to this practice, and where physicians were once a bulwark of scepticism against any trading on credulousness, they are now the most cost-effective marketing tool companies have.

Mongering applies to conditions from mild elevations of blood pressure or lipids, to bone thinning. No one argues hypertension or hypercholesterolaemia are not real or that in malignant cases these conditions do not constitute valid targets of treatment. But malignant cases are rare. In cases that are not malignant, when the likely intervention is with a toxic compound rather than a proposed alteration of lifestyle, there is or should be a boundary.

Psychiatry was once plagued by “boundary violations”, where physicians exploited the dependence of their patients. All the indications are that we are now in a new era of drug-related boundary violations. There is perhaps nowhere in medicine where this is more obvious than in the case of bipolar disorders, with adults treated with bizarre cocktails and children put on some of the most lethal drugs in medicine.

Making it clear that the term “mood stabilizer” is itself an advert and that the notion of bipolar disorder can be viewed as an instance of rebranding does not deny the reality of anything. The key concerns are not reality in this sense, but rather when to treat. As the history of hysteria shows, the best pseudo-convulsions come from patients with convulsive disorders, and the most realistic somatization from patients with other real disorders. Patients conform their presentations to the interests of their doctors. Drug companies know this. Patients deserve physicians alert to such possibilities. In the current welter of bipolar presentations, one worry is that patients with severe manic-depressive disorder will lose out. Another is that research on this most difficult of disorders will be invalidated by a dilution by patients with other problems. A final worry is that when the marketing caravan moves on, manic-depressive illness will be left once more under-resourced, and researchers will have one less lever to pull as they have “had their chance”.
